# A Legacy of Impact in Global Health

**DOI:** 10.4269/ajtmh.24-0357

**Published:** 2024-07-16

**Authors:** Mark L. Eberhard

**Affiliations:** Retired, Division of Parasitic Diseases and Malaria, CDC, Atlanta, Georgia

This supplemental issue with the *American Journal of Tropical Medicine and Hygiene* is being published to recognize and honor former U.S. President Jimmy Carter and First Lady Rosalynn Carter for their longstanding and tireless contributions to public health.^1^ Co-founded by the Carters in 1982, The Carter Center focuses its efforts on central values of peace, human rights, and alleviation of suffering. Because of the core belief that health is a human right, The Carter Center developed health programs (encompassing mental health and neglected tropical diseases [NTDs]) in parallel with its peace programs (encompassing conflict resolution and democracy).

The Carters grew up in rural southern Georgia and knew first-hand the impacts that infectious diseases and mental illness had on impoverished people, and how they compounded inequities. The fact that President Carter’s mother, Miss Lillian, was a nurse, instilled in him at an early age the understanding that good health care was essential to improve people’s lives. Rosalynn Carter’s life-long promotion of mental health began when she was First Lady of Georgia in the 1970s and continued through the White House years to her work at The Carter Center.

When establishing the Center’s programs, President Carter recruited William (Bill) Foege from the U.S. Centers for Disease Control and Prevention (CDC), where he was Director, to be the Center’s first Executive Director. Dr. Foege was one of the world’s most respected public health leaders and known for his leadership role in smallpox eradication. Dr. Foege and President Carter agreed that The Carter Center should work in health, and especially on eradication efforts, because disease eradication offered the ultimate form of public health equity.^2^ For eradication to be successful, no one can be left behind, regardless of their circumstance. Efforts were accompanied by the notion that, if the outcome had sufficient merit, a certain degree of risk of failure was acceptable and should not dissuade The Carter Center from undertaking the endeavor. Carter and Foege both recognized that eradication is not only equitable but provides a lasting impact.

The Guinea Worm Eradication Program (GWEP) became the flagship health program at The Carter Center in 1986. Dr. Foege recruited Dr. Donald Hopkins, who served as Director of Center health programs for almost thirty years and continues as a special advisor. Over the years, dozens of individuals from CDC have undertaken short- or long-term assignments in the GWEP and four other Carter Center NTD programs that came after, addressing onchocerciasis, lymphatic filariasis, trachoma, and schistosomiasis. This close association of both people and programs between The Carter Center and CDC continues today, is highly advantageous for both institutions,^3^ and is complimented by close collaboration with Rollins School of Public Health of Emory University and the Task Force for Global Health.

**Figure 1.**
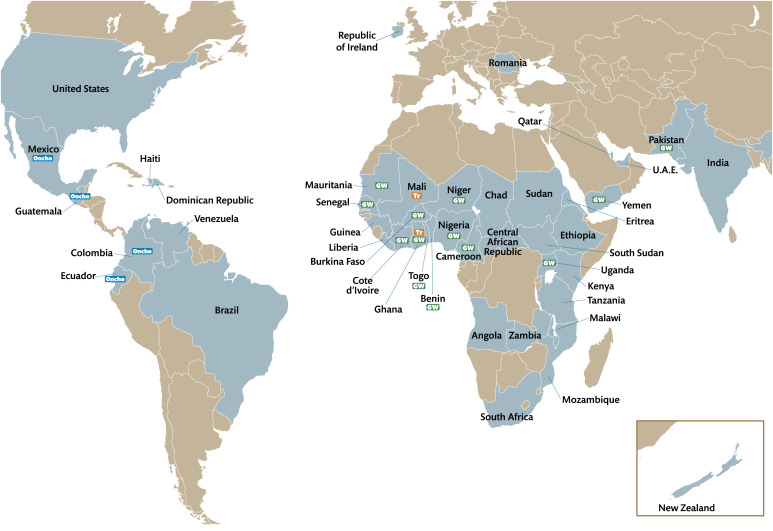
Countries that have received Carter Center health program assistance past and present are represented in light blue. The Carter Center assisted countries which have been WHO certified as eradicating dracunculiasis transmission (GW), WHO verified as eliminating onchocerciasis transmission (Oncho), or WHO validated as trachoma no longer being a public health problem (Tr). Note that India, Kenya, CAR, and DRC have been WHO certified as eradicating dracunculiasis, but those countries did not receive direct Carter Center assistance. Map courtesy of The Carter Center.

**Figure 2.**
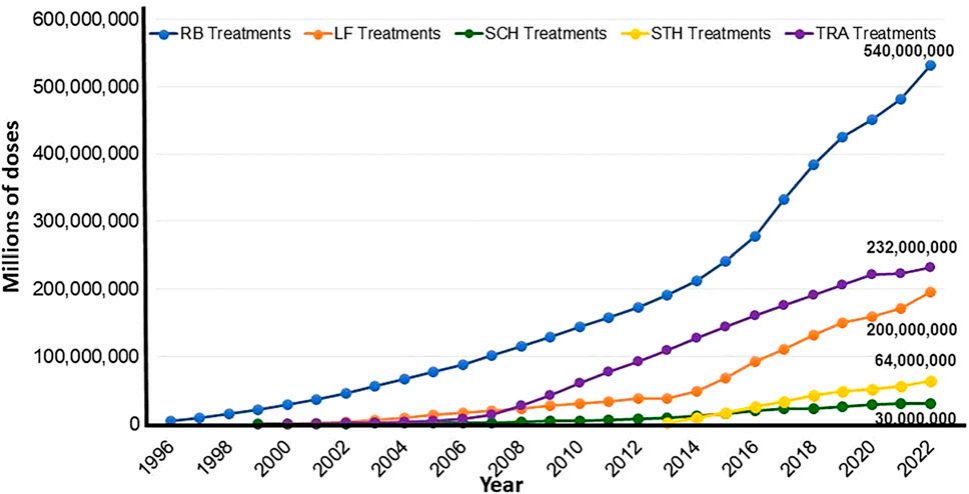
The Carter Center health program’s cumulative treatments 1996–2022 for river blindness, trachoma, lymphatic filariasis, schistosomiasis, and soil-transmitted helminths illustrating the effective scale-up of programs. Data courtesy of The Carter Center.^5^^,^^6^

**Figure 3.**
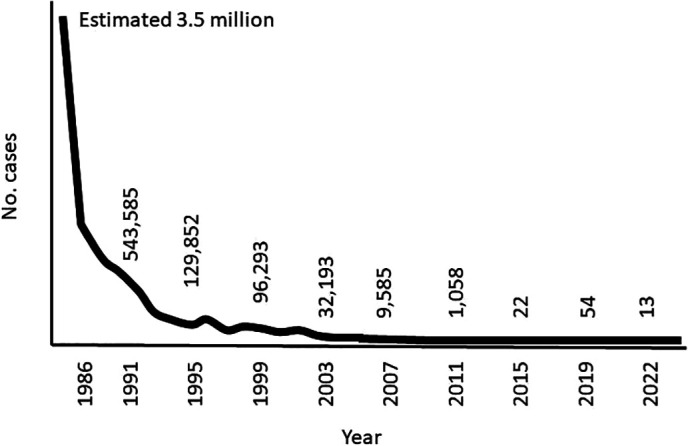
Human Guinea worm cases 1986–2022 showing the impact of a highly successful program across numerous countries. In 1989, the first year of national case searches, there were nearly 900,000 cases enumerated.

On a global scale, in alignment with WHO 2030 goals^4^ and working with a network of partners, including WHO, ministries of health, drug companies, donors and foundations, political and opinion leaders, public health scientists, and affected communities, the Carters lent their considerable advocacy and fund-raising abilities to invigorate global and national NTD programs. These programs were largely ignored and underfunded but had extremely lofty goals – more often than not looking to eliminate or eradicate disease. The programs,^5^ a legacy of President and Rosalynn Carter, have been hugely successful, and are active, thriving, expanding, and improving the lives of millions in over 38 countries (Figure 1). A brief overview of each provides some measure of their impact and success (Box 1; Figures 2 and 3).

Box 1 **Carter Center health programs and their impact^4^**

Mental Health^6^: Since its inception in 1982, The Center has worked to improve mental health in the U.S. by promoting better public policy that puts mental health on par with physical health. The Rosalynn Carter Journalism Fellowship program has equipped 250 journalists from eight countries to thoughtfully and accurately cover the global mental health crisis and build public support for policy change. The Mental Health program expanded its international work in 2009 by training mental health clinicians in Liberia, and aims to train clinicians in four new countries by 2027.

Dracunculiasis (Guinea worm disease)^7^: In 1986, the Center began leading the global Guinea Worm Eradication Program. Together with the CDC, WHO, UNICEF, and other partners, the Center grew the campaign to full scale by assisting endemic countries to conduct national case searches, implement interventions, monitor impact, and prepare countries for certification. Two major interventions, the use of Abate and nylon cloth filters, were greatly assisted by the donations of materials by American Cyanamid, BASF, DuPont Corporation, and Vestagaard. The global burden of dracunculiasis has been reduced from an estimated 3.5 million human cases annually in 21 countries in 1986 to 14 cases in a few countries in 2023^3^, averting more than 80 million cases. To date, 17 countries have received WHO certification of elimination of transmission.^1^ The Center looks to eradicate dracunculiasis by 2030.

The International Task Force for Disease Eradication (ITFDE)^8^: Formed in 1998, the ITFDE brings together leading global health experts to advise on the potential for future disease eradication and control and evaluate current research and findings. Over the last two decades, the ITFDE has held 33 meetings, discussed 22 diseases, and made 244 recommendations. Eight diseases (dracunculiasis, lymphatic filariasis, mumps, poliomyelitis, rubella, *Taenia solium* cysticercosis, yaws, and measles) have been identified as potentially eradicable.

Onchocerciasis (river blindness)^9^: In 1996, this program was launched by assuming activities of the River Blindness Foundation in six Latin American countries aiming to eliminate transmission (Brazil, Colombia, Ecuador, Guatemala, Mexico, and Venezuela), and disease control in Africa (Ethiopia, Nigeria, Cameroon, Sudan, and Uganda). In 2013, together with countries, a critical decision was made to transition the Carter Center’s assistance to African onchocerciasis programs from control to elimination of transmission in areas where The Carter Center worked with national programs. The Center has assisted in delivery of over 500 million doses of ivermectin (Mectizan©, donated by Merck & Co), helping to eliminate transmission (as verified by WHO) in four Latin American countries (Colombia, Ecuador, Mexico and Guatemala).^1^ These four countries were the first ever verified as having eliminated transmission country-wide. In addition, transmission has been interrupted in numerous Carter Center assisted foci in Africa, including in Uganda, Ethiopia, Sudan, and Nigeria, proving that elimination of onchocerciasis can be accomplished even in Africa. This has resulted in over 30 million people no longer being treated for onchocerciasis. The Center aims to eliminate transmission in the Americas and Uganda by 2030 and from Carter Center assisted areas in Ethiopia, Nigeria, and Sudan by 2035.

Trachoma^10^: In 1998, President Carter launched the Center’s Trachoma Control Program to battle the world’s leading cause of preventable infectious blindness. The program has worked in Mali, Ethiopia, Sudan, South Sudan, Niger, Nigeria, Ghana, and Uganda. Cumulatively, through 2022, the Center has assisted ministries of health to provide 902,363 persons with eyelid surgeries, distribute 232 million doses of antibiotics^2^ (azithromycin (Zithromax^TM^-donated by Pfizer) or tetracycline eye ointment), and constructed 3.6 million latrines. An estimated 65 million people are no longer at risk for trachoma in Carter Center assisted areas. Trachoma has been eliminated as a public health problem from Ghana and Mali and is on track for elimination from the other countries by 2030.

Schistosomiasis^9^: Since 1999 the Carter Center has assisted areas in Nigeria and successfully controlled high intensity infections in school aged children with schistosomiasis (by distributing 62 million cumulative praziquantel treatments, most of which were donated by E-Merck) and soil-transmitted helminth infection (with 30 million treatments of either mebendazole donated by Johnson and Johnson or albendazole donated by GSK).^2^

Lymphatic filariasis^9^: In 2000, lymphatic filariasis mass treatment began in Nigeria with combined ivermectin (donated by Merck) and albendazole (donated by GSK)^2^ and distribution of long lasting insecticidal nets for LF and malaria control in Nigeria, and later in Ethiopia. Over 24 million people no longer need treatment for LF in Carter Center assisted areas in Nigeria and Ethiopia; two states in Nigeria have eliminated transmission. Carter Center assisted areas in Ethiopia and Sudan are targeting elimination of lymphatic filariasis by 2030.

Hispaniola Initiative^11^: In 2006, ITFDE made the recommendation to eliminate malaria and lymphatic filariasis from their last focus in the Caribbean, the island of Hispaniola, shared by the Dominican Republic and Haiti. Subsequently, President Carter, with the heads of state from both countries, initiated a pilot program in 2008 to accelerate the elimination of these two mosquito-borne infections from Hispaniola. The program has expanded island wide, with the Center assisting the Dominican Republic to eliminate transmission of lymphatic filariasis and secure WHO verification by 2025. Elimination of lymphatic filariasis from Haiti, and malaria from the entire island, is targeted by 2030. The initiative has provided lessons on cross border collaboration despite political challenges and natural disasters.

Civil strife, turmoil, other unexpected developments, and even Mother Nature encumber most, if not all, programs working in low- and middle-income countries, and The Carter Center’s are no exception. Reaching remote communities “beyond the end of the road” that are often excluded, as well as marginalized indigenous or minority groups that have special needs, is especially challenging. Nonetheless, the dedication and perseverance of The Carter Center staff, following the example of President and Mrs. Carter have found ways to motivate politicians, governments, and local communities alike to get needed interventions to those most in need. The Carters did not just talk the talk, they walked the walk. For decades, they made repeated trips to affected countries, stopping not only in the capital cities to meet and engage state leaders, but traveling (often with those same leaders) to remote affected communities where they were able to see firsthand the difficulties and accomplishments of various programs. President Carter also wrote numerous letters and made telephone calls to heads of state and heads of organizations advocating not only for Carter Center programs, but for global campaigns. President Carter even negotiated a temporary cease fire in Sudan, in 1995, that brought relief to millions of people for almost 6 months and permitted critical Guinea worm program work to proceed.^12^ This degree of engagement and understanding of specific aspects of each disease led to inspiration and motivation across programs within The Carter Center and among external partners, reaching even the most remote country staff. Such commitment to optimism and excellence kept programs moving forward through the most difficult of times.

These challenges have required ingenious modification of strategies based on practical field experiences, while always remaining focused on data to monitor progress toward desired outcomes. The Carter Center is not a research organization, but a proactive, boots-on-the-ground implementing partner with afflicted communities, ministries of health, and WHO. Yet, a research agenda has always existed in Carter Center programs in association with active public health interventions with the goal of rapidly identifying and deploying new ways to solve problems, and help programs expand and function better. The 16 articles contained in this supplement highlight outcomes of such research in each health program currently underway at The Carter Center. The articles add to an already large body of publications by Carter Center personnel and their colleagues, consisting of over 300 peer-reviewed articles. At its heart, this body of literature, which now includes this Supplement, further advances the work, whether it be best practices, new and better targeted interventions, testing and incorporation of new assays to assess transmission interruption, basic research, or monitoring and improving of the situation “on the ground”.

Looking towards 2030, The Carter Center plans to continue expansion of its programs while strengthening linkages between its health and peace programs to help people living in insecure areas live healthier, more peaceful lives. Further, The Carter Center hopes to explore new ways to integrate mental health and NTD work, addressing the despair and suffering that arise from chronic illness and stigma. Lastly, The Carter Center looks to strengthen grass roots health system outreach, working toward the control, elimination, and ultimately eradication of many of the world’s most dreadful NTDs.
